# Comparing audiovisual visit and in-person visit note quality

**DOI:** 10.1093/jamiaopen/ooag014

**Published:** 2026-04-29

**Authors:** Harry B Burke, Albert Hoang, Heidi King, Joseph Benich, Brandon Brown, Christopher Bunt, Sean Haley, Paul Hemmer, William Kelly, Renee Mallory, Louis Pangaro, Wendy Shen, Viktoria Gagarin

**Affiliations:** George Washington University School of Medicine, Washington, DC, 20037, United States; George Washington University School of Medicine, Washington, DC, 20037, United States; George Washington University School of Medicine, Washington, DC, 20037, United States; Defense Health Agency, Falls Church, VA, 22040, United States; George Washington University School of Medicine, Washington, DC, 20037, United States; Medical College of South Carolina, Charleston, SC, 29401, United States; George Washington University School of Medicine, Washington, DC, 20037, United States; Medical College of South Carolina, Charleston, SC, 29401, United States; George Washington University School of Medicine, Washington, DC, 20037, United States; George Washington University School of Medicine, Washington, DC, 20037, United States; Medical College of South Carolina, Charleston, SC, 29401, United States; George Washington University School of Medicine, Washington, DC, 20037, United States; George Washington University School of Medicine, Washington, DC, 20037, United States; George Washington University School of Medicine, Washington, DC, 20037, United States; George Washington University School of Medicine, Washington, DC, 20037, United States; George Washington University School of Medicine, Washington, DC, 20037, United States; College of Medicine, University of Iowa, Iowa City, IA, 522040, United States; George Washington University School of Medicine, Washington, DC, 20037, United States

**Keywords:** audiovisual visit, in-person visit, clinical quality, virtual visit, virtual health, telemedicine, telehealth, clinical note, QNOTE, quality of care, outpatient visit, patient safety, physician-patient interaction

## Abstract

**Background:**

The pandemic dramatically increased in the frequency of audiovisual medical visits and the rate of audiovisual visits remains higher than before the pandemic. These visits have the potential to be an important clinical modality. Researchers have assessed several aspects of audiovisual visits but they have not investigated their clinical note quality. The goal of this study is to determine the quality of audiovisual and in-person clinical notes and to compare their quality.

**Methods:**

From a population of 1660 established outpatient primary care type 2 diabetic patient visits occurring in 2021 we randomly selected 100 audiovisual and 100 in-person visits. QNOTE, a validated instrument that measures clinical note quality, was used by 7 experienced primary care physicians to assess 4 key elements of the clinical note: the chief complaint, history of present illness, assessment, and plan.

**Results:**

The mean quality scores (out of 100) and their standard deviations (SD) were: overall, audiovisual 75.8 (SD 9.6), in-person 86.5 (SD 4.4), *P* < .0001; chief complaint, audiovisual 72.4 (SD 23.0), in-person 83.0 (SD 14.7), *P* < .0001; history of present illness, audiovisual 63.7 (SD 22.9), in-person 82.5 (SD, 15.8), *P* < .0001; assessment, audiovisual 82.9 (SD 14.8), in-person 89.8 (SD 10.0), *P* = .0002; and plan, audiovisual 84.1 (SD 13.7), in-person 90.9 (SD 8.7), *P* < .0001. The in-person scores are consistent with a previous QNOTE study. The rater intraclass correlation coefficient was excellent (0.81, 95% CI, 0.76-0.86).

**Conclusion:**

Audiovisual visits demonstrated a lower note quality than in-person visits. To our knowledge, this is the first study to assess the quality of audiovisual visit notes and compare them to the quality of in-person visit notes.

## Introduction

Historically, the physician-patient interaction was conducted face-to-face. The advent of the telephone allowed remote auditory interactions (telehealth, telemedicine), while the internet gave rise to email and, more recently, remote audiovisual interactions. Until the COVID-19 virus pandemic, audiovisual interactions were relatively rare, accounting for less than 1% of all physician-patient visits.^1^^,^^2^ During the pandemic, the frequency of audiovisual visits increased dramatically.^3–8^ Within the first 4 months, audiovisual visits increased to over 40% of all primary care visits.^1^^,^^2^ The increase in remote interactions was driven by the need to reduce the transmission of the virus, changes in clinician outpatient staffing and appointment schedules, and patient preferences.^3^^,^^9^^,^^10^ Although the incidence of the COVID-19 virus has subsided, audiovisual visits have remained at a higher relative and absolute rate compared to pre-pandemic levels^8^ and audiovisual visits now play an important role in patient care. Researchers have assessed several aspects of audiovisual visits but they have not investigated the clinical note quality of the visits. Our aim is to assess audiovisual clinical note quality and to compare it to in-person note quality.

Physicians’ notes document the clinical activities that occur during physician-patient interactions. Using QNOTE,^11^^,^^12^ a validated instrument that measures clinical quality, we assess the quality of audiovisual visit notes and compare it to the quality of in-person notes. To our knowledge, this is the first study to assess the quality of audiovisual notes and to compare them to the quality of in-person notes.

## Methods

We selected 1660 established outpatient primary care visits of type 2 diabetic patients within the military health system (MHS) in 2021. Research has shown that MHS patients and their care are similar to that of the general population.^13–16^ A person unaffiliated with the study removed inaccurately coded visits and notes that only consisted of referrals, refills, and preoperative clearances. They de-identified the remaining notes and removed any mention of the type of visit. One hundred audiovisual and 100 in-person visit notes were randomly selected, copied, and pasted into individual Word documents (one patient visit per document) and the 200 notes were randomized.

QNOTE, a widely recognized and validated instrument for measuring clinical note quality, was used for this study.^11^^,^^12^ We focused on 4 key note elements: the chief complaint, history of present illness, assessment, and plan. The element evaluation criteria were: chief complaint, was there was sufficient information to direct the history of present illness and did it include the pertinent details; history of present illness, was there sufficient information, and was it concise, clear, and well organized; assessment, were the problems prioritized in their order of importance, was there sufficient information, and was it clear and concise; and plan, were the goals and objectives prioritized in their order of importance, was there sufficient information, and was it clear and concise. Seven general internal medicine and family medicine physicians, each with at least 5 years of post-residency clinical experience, used QNOTE to assess the 4 note elements. They were instructed to use their clinical judgment in evaluating the quality of the notes. No training was provided and none of the physicians requested additional information. For every note, the physician reviewers rated each of the 4 note elements in terms of their being “acceptable, partially acceptable, or unacceptable,” with scores of 100, 50, and 0, respectively. The overall score was the average of the element scores.

The number of words in each element was calculated by analyzing each sentence to determine whether it contained clinical information. Sentences with clinical information were labeled as clinical sentences and their words were counted as clinical. Sentences without clinical information were labeled as administrative and their words were counted as administrative. Continuous variables were assessed using the Student’s t-test (2-tailed), categorical variables with the chi-square test, and rater agreement was assessed using the intraclass correlation coefficient (SAS, Cary, NC, United States).

## Results

The mean quality scores (out of 100 possible points) and their standard deviations (SD) were: overall, audiovisual 75.8 (SD 9.6), in-person 86.5 (SD 4.4), *P* < .0001; chief complaint, audiovisual 72.4 (SD 23.0), in-person 83.0 (SD 14.7), *P* < .0001; history of present illness, audiovisual 63.7 (SD 22.9), in-person 82.5 (SD, 15.8), *P* < .0001; assessment, audiovisual 82.9 (SD 14.8), in-person 89.8 (SD 10.0), *P* = .0002; and plan, audiovisual 84.1 (SD 13.7), in-person 90.9 (SD 8.7), *P* < .0001 (Table 1). Audiovisual visits demonstrated lower clinical quality scores than in-person visits. The rater intraclass correlation coefficient was 0.81 (95% CI, 0.76-0.86).

**Table 1.** Comparison of in-person and audiovisual rater scores by element.

Note elements	**Audiovisual** (mean, SD)	**In-person** (mean, SD)
Chief complaint^a^	72.4 (23.0)	83.0 (14.7)
History of present illness^a^	63.7 (22.9)	82.5 (15.8)
Assessment^b^	82.9 (14.8)	89.8 (10.0)
Plan^a^	84.1 (13.7)	90.9 (8.7)

Comparing in-person to audiovisual.

a *P* < .0001,

b *P* = .0002.

The audiovisual chief complaint and history of present illness scores were significantly different, *P* = .0081, but the audiovisual assessment and plan scored similarly. The in-person chief complaint and history of present illness, and the assessment and plan, all scored similarly. The audiovisual chief complaint and history of present illness scores were significantly lower than the assessment and plan scores, *P* < .0001. The in-person chief complaint and history of present illness scores were significantly lower than the assessment and plan scores, *P* < .0001.

**Figure 1.**
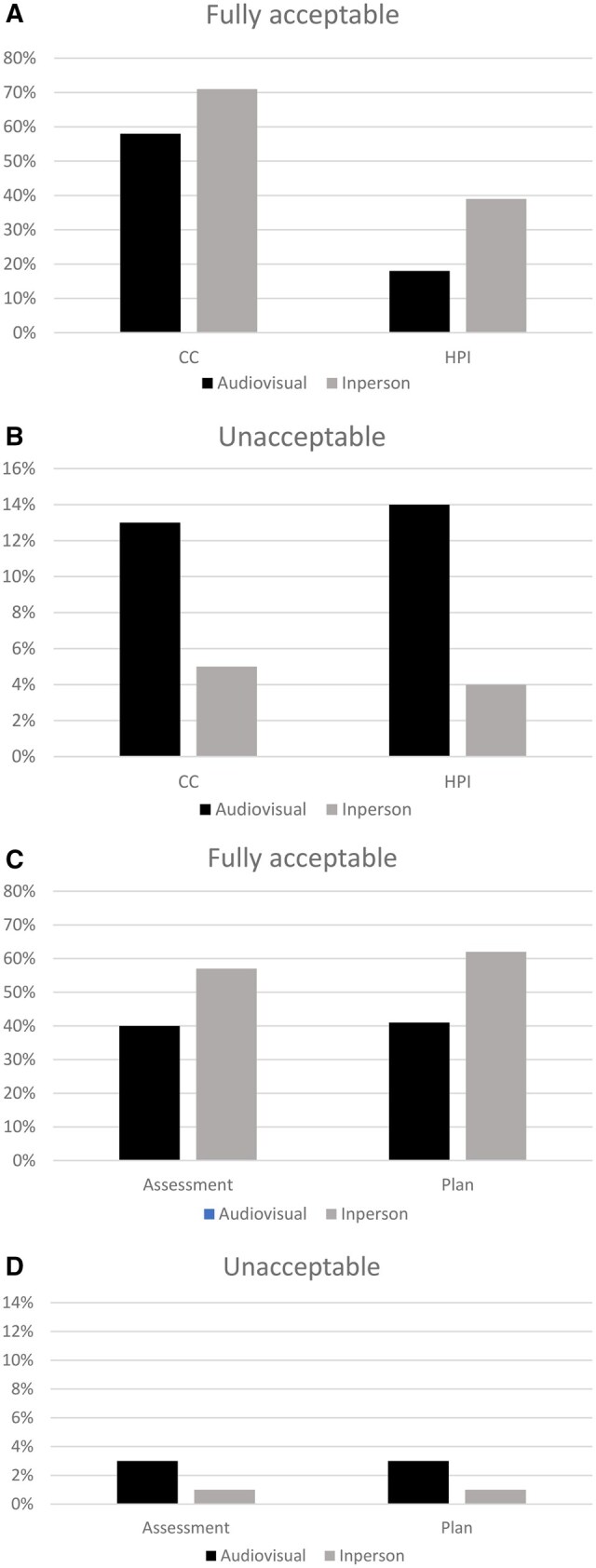
(A) Comparison of audiovisual and in-person notes in terms of the percentage of chief complaint (CC) and history of present illness (HPI) elements deemed fully acceptable to the reviewers. (B) Comparison of audiovisual and in-person notes in terms of the percentage of chief complaint (CC) and history of present illness (HPI) elements deemed unacceptable to the reviewers. (C) Comparison of audiovisual and in-person notes in terms of the percentage of assessment and plan elements deemed fully acceptable to the reviewers. (D) Comparison of audiovisual and in-person notes in terms of the percentage of assessment and plan elements deemed unacceptable to the reviewers.

In order for a note to be fully acceptable, the physician had to collect the appropriate information, provide the correct diagnosis and treatment plan, and properly report this information in the note. The frequency of note element acceptability/unacceptability for the chief complaint and history of present illness by visit type is shown in Figure 1A and B. In terms of the chief complaint (Figure 1A), 58% of audiovisual and 71% of in-person notes were considered fully acceptable. In terms of the history of present illness, 18% of audiovisual and 39% of in-person notes were scored fully acceptable. For the unacceptable elements (Figure 1B, note the change in the *y*-axis), in terms of the chief complaint, 13% of audiovisual and 5% of in-person notes were scored unacceptable. In terms of the history of present illness, 14% of audiovisual and 4% of in-person notes were scored unacceptable. Compared to the audiovisual chief complaint and history of present illness, the in-person chief complaint was more often considered fully acceptable and rarely unacceptable, *P* = .013, and the in-person history of present illness was also more often considered fully acceptable and rarely unacceptable, *P* < .0001.

The frequency of note element acceptability/unacceptability for the assessment and plan by visit type is shown in Figure 1C and D. In terms of the assessment (Figure 1C), 40% of audiovisual and 57% of in-person notes were considered fully acceptable. In terms of the plan, 41% of audiovisual and 62% of in-person notes were considered fully acceptable. For unacceptable elements (Figure 1D, note the change in the *y*-axis), in terms of the assessment, 3% of audiovisual and 1% of in-person notes were considered unacceptable. In terms of the plan, 3% of audiovisual and 1% of in-person notes were considered unacceptable. Compared to the audiovisual assessment and plan, the in-person assessment was more often considered fully acceptable and rarely unacceptable, *P* = .0007, and the in-person plan was also more often considered fully acceptable and rarely unacceptable, *P* < .0001.

Comparing total words and clinical words, in terms of the chief complaint, the number of words in the audiovisual notes, mean 5.7 (SD 3.7), and the number of words in the in-person notes, mean 5.3 (SD 2.1), and they were not significantly different. For the history of present illness (Table 2), the audiovisual total words, mean 171 (SD 115), and the in-person total words, mean 134 (SD 80), were significantly different, *P* < .0001. The audiovisual clinical words, mean 77 (SD 67), and the in-person clinical words, mean 116 (SD 77), were significantly different, *P* < .0001. The audiovisual total words, mean 171 (SD 115), and the audiovisual clinical words, mean 77 (SD 67), were significantly different, *P* < .0001. The in-person total words, mean 134 (SD 80), and the in-person clinical words, mean 116 (SD 77), were not significantly different.

**Table 2.** For the history of present illness, a comparison of audiovisual and in-person visits in terms of the total number of words and number of clinical words.

Note elements	**Total words** ^a^ (mean, SD)	**Clinical words** ^a^ (mean, SD)
Audiovisual^a^	171 (115)	77 (67)
In-person^b^	134 (80)	116 (77)

a Comparing in-person to audiovisual, total words, *P* < .0001.

b Comparing in-person, total words to clinical words, NS.

For the assessment and plan (Table 3), the audiovisual total words, mean 222 (SD 162), and the in-person total words, mean 290 (SD 163), were significantly different, *P* = .0035. The audiovisual clinical words, mean 193 (SD 154), and the in-person clinical words, mean 266 (SD 161), were significantly different, *P* = .0013. The audiovisual total words, mean 222 (SD 162), and the audiovisual clinical words, mean 193 (SD 154), were not significantly different. The in-person total words, mean 290 (SD 80), and the in-person clinical words, mean 266 (SD 161), were not significantly different.

**Table 3.** For the assessment and plan, a comparison of the audiovisual and in-person visits in terms of the total number of words and number of clinical words.

Note elements	**Total words** ^a^(mean, SD)	**Clinical words** ^b^(mean, SD)
Audiovisual^c^	222 (162)	193 (154)
In-person^d^	290 (163)	266 (161)

a Comparing audiovisual to in-person, total words, *P* = .0035.

b Comparing audiovisual to in-person, clinical words, *P* = .0013.

c Comparing audiovisual, total to clinical words, NS.

d Comparing in-person, total words to clinical words, NS.

One must be careful when assessing specific aspects of clinical notes. For example, if one searched the audiovisual notes for “patient agreement,” one would find that 50% of the audiovisual notes possessed patient agreement but only 41% of the in-person notes reflected patient agreement (Table 4). But the audiovisual “patient agreement” had 2 meanings. It meant that the patient agreed to the audiovisual visit and it meant that there was medical agreement. Manually re-reviewing patient agreement in each note, we fund that the audiovisual medical agreement of 43% was not significantly different from the in-person medical agreement of 41%. It is noteworthy that less than half the notes contained an acknowledgement of patient medical agreement.

**Table 4.** Quality study audiovisual visit: types of patient agreement.

Audiovisual visit agreement only	7%
Medical care agreement only	3%
Both audiovisual and medical	40%
Neither audiovisual nor medical (none)	50%

Except for 3 encounters, all the audiovisual and in-patient visits were coded as either 99213 or 99214. Seventy-six percent of the audiovisual notes were coded 99213 and 24% were coded 99214. Forty-six percent of the in-person notes were coded 99213 and 54% were coded 99214. There were no significant differences between the audiovisual 99213 quality scores and the 99214 quality scores and there were no significant differences between the in-person 99213 quality scores and the 99214 quality scores.

## Discussion

We assessed the note quality of audiovisual visits and in-person visits. We found that audiovisual visits demonstrated a lower note quality than in-person visits. The scores of the in-person chief complaint, history of present illness, assessment, and plan were consistent with those reported in the previous QNOTE study.^2^ The rater intraclass correlation was excellent and it was similar to the result of the previous QNOTE study.^2^

In order for a note to be fully acceptable, the physician had to collect the appropriate information, provide the correct diagnosis and treatment plan, and properly report this information in the note. The significantly greater rate of fully satisfactory in-person notes suggests that in-person visits may have been a higher clinical quality than audiovisual visits.

Are more words in a note related to better quality? We calculated the word count for both audiovisual and in-person notes (Table 4). We found that, in terms of total word count, the history of present illness section of audiovisual notes was significantly longer than that of in-person notes. However, after removing non-clinical information, the audiovisual notes actually contained significantly less clinical content. The higher word count in the history of present illness for audiovisual notes was primarily due to administrative content, such as boilerplate text indicating that the interaction as an audiovisual visit. Furthermore, during audiovisual visits, the physician and patient often focus on audiovisual-specific administrative tasks and address equipment and connectivity issues, which reduces the time available for clinical information gathering and medical reasoning.^11^^,^^17^ For a fixed visit duration, there is a tradeoff between administrative/technical activities and clinical activities.^18^

Audiovisual visits tend to require more follow-up care than in-person visits. Reed et al.^19^ found that follow-up visits within 7 days were more common after audiovisual visits (6.2%) than in-person visits (1.3%). Graetz et al.^20^ also fund that return visits were more frequent following audiovisual visits (5.3%) compared to in-person (1.2%). However, in palliative care, there was no difference in the quality-of-life scores at 24 weeks between patients receiving audiovisual or in-person visits.^21^ Additionally, for patients with heart failure, 7-day all-cause hospitalization rates did not differ between audiovisual and in-person visits.^22^

The physical examination has historically been seen as a barrier to remote health interactions,^20^ but the development of mobile electronic technologies for home-based physical data acquisition has diminished these concerns. Technologies, including devices and apps for ophthalmology and dermatology, thermometers, stethoscopes, blood pressure monitors, scales, pulse oximetry, app-based electrocardiograms, point-of-care ultrasound, and others have become prevalent in healthcare. Some of these technologies can supplant in-person physical examinations. They can also provide real-time monitoring which can alert physicians to acute and emergent clinical issues. A scoping review of 25 publications found that the most common virtual examinations were of the musculoskeletal system, the head and neck, and the chest and that both physicians and patients found the virtual examination to be useful.^23^ Of note, virtual physical examination challenges include technological and financial issues, that some types of medical conditions are not amiable to virtual examinations, and that patients’ limited cognitive and physical capabilities may preclude their participation in an audiovisual visit. On the other hand, many diagnostic, treatment, and follow-up interactions are suitable for virtual visits.^24^

Audiovisual visits can remove barriers to care, for example, they can be very useful for patients who have problems accessing care, such as elderly patients, for those living far from a medical facility, for patients with limited travel options, for those who work during the day, very ill patients, and patients with disabilities.^20^^,^^25^ Audiovisual visits increase convenience, for example, on average, older adults spend 3 weeks a year receiving ambulatory care outside their home. And audiovisual visits can reduce this time requirement.^27^ Audiovisual visits can increase the overall number of visits^20^^,^^26^ and reduce no-show rates. Moreover, they can benefit low-resource working patients who cannot afford to take time off from work to visit a clinic.

Several issues can affect the success of audiovisual visits. Few clinicians—and even fewer patients—have received systematic training for audiovisual interactions.^11^^,^^27^ At least 20% of patients experience technical issues.^17^^,^^26^^,^^28^^,^^29^ Communication can be challenging in audiovisual settings because the participants interact with a 2-dimensional computer screen which limits their ability to detect paralanguage and nonverbal cues^30^^,^^31^ and it creates a social separation.^32^ Additionally, audiovisual visits are often scheduled for the same duration as in-person visits, even though they may require more time due to technical and administrative considerations and to interpersonal communication constraints. Furthermore, audiovisual visits can engender safety issues, including medical errors such as missed or incorrect communication, diagnostic or treatment delays, incomplete documentation, and medication errors,^33^ all of which can result in patient harm.^34–37^ Of course, these issues also arise in in-person visits.

Recommendations for improving audiovisual visits include (1) recognizing them as full-fledged clinical encounters, (2) establishing patient selection criteria, (3) providing training for both physicians and patients regarding how to perform an audiovisual interaction, (4) allowing sufficient time for audiovisual visits, (5) creating administrative policies and procedures that support audiovisual visits, and (6) conducting research on how to improve the quality and safety of audiovisual visits.

Physicians’ notes document the clinical activities that occur during physician-patient interactions. This record is fundamental to safe, high quality medical care. Audiovisual visits demonstrated a lower note quality than in-person visits. The significantly greater rate of fully satisfactory in-person notes suggests that in-person visits may have been a higher clinical quality than audiovisual visits. However, we believe that with the appropriate patient selection, adequate physician and patient education, the necessary equipment, and sufficient administrative support—including allowing sufficient time for each visit—audiovisual visits can be an important clinical modality. To our knowledge, this is the first study to assess the quality of audiovisual visit notes and to compare them to the quality of in-person visit notes.

## Data Availability

Clinical data cannot be shared.
